# Real‐time bowel sound analysis using newly developed device in patients undergoing gastric surgery for gastric tumor

**DOI:** 10.1002/jgh3.12515

**Published:** 2021-02-26

**Authors:** Tsutomu Namikawa, Sachi Yamaguchi, Kazune Fujisawa, Maho Ogawa, Jun Iwabu, Masaya Munekage, Sunao Uemura, Hiromichi Maeda, Hiroyuki Kitagawa, Michiya Kobayashi, Kenichi Matsuda, Kazuhiro Hanazaki

**Affiliations:** ^1^ Department of Surgery Kochi Medical School Nankoku Kochi Japan; ^2^ Department of Human Health and Medical Sciences Kochi Medical School Nankoku Kochi Japan; ^3^ Department of Emergency and Critical Care Medicine, Faculty of Medicine University of Yamanashi Chuo Yamanashi Japan

**Keywords:** bowel sound analysis, gastric cancer, intestinal peristalsis, surgical stress

## Abstract

**Background and Aim:**

Objective measurements are not available for determining bowel sounds. The present study sought to evaluate the efficacy of a novel bowel sound monitoring system for perioperative use in patients undergoing gastric surgery.

**Methods:**

The study enrolled 14 patients who underwent surgery for gastric cancer at Kochi Medical School from 2017 to 2018. Preoperative and postoperative bowel sounds were recorded using a newly developed real‐time analysis system in the operating theater and recovery room. Clinical information and bowel sound count data were obtained to compare preoperative and postoperative measures.

**Results:**

The median preoperative and postoperative bowel sound counts across all patients were 1.4 and 2.5 counts per minute (cpm), respectively. In patients who underwent laparoscopic gastrectomy, the postoperative bowel sound count was significantly higher than that recorded preoperatively (2.3 vs. 1.6 cpm, *P* = 0.005). The findings also revealed a significant negative correlation between postoperative bowel sound count and operation time (*r* = −0.714, *P* = 0.003).

**Conclusions:**

The real‐time bowel sound analysis system tested herein presents a promising diagnostic tool to quantitatively evaluate bowel movements associated with surgery. Our results suggested a need for shorter operation times for gastric procedures with respect to peristalsis recovery and supported the use of minimally invasive surgery.

## Graphical abstract

We prospectively evaluated bowel sound counts using newly developed analysis system in patients who underwent gastric surgery. A real‐time bowel sound analysis system is promising as a diagnostic tool to quantitatively evaluate bowel movement. In this study, analysis of bowel sounds in patients undergoing gastrointestinal surgery suggested a need for shorter operation times from the aspect of peristalsis recovery and supporting the use of minimally invasive surgery.

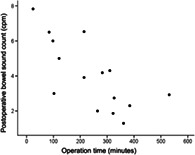

## Introduction

Contractions of the alimentary tract and mixing of the gaseous and liquid contents together generate bowel sounds. Traditional auscultation of bowel sounds is a basic technique that forms part of a physical abdominal assessment to determine whether normal bowel sounds are present and as a diagnostic aid for gastrointestinal diseases;^1^ however, the results are generally too empirical and subjective, limited by the physician's skill, and difficult to precisely document and reassess.

Objective measurements are not available for determining bowel sounds, and there is a distinct lack of published clinical research to support any discussion on the value of auscultation.^1^, ^2^ Indeed, previous studies demonstrate the need for improved training, education, and an understanding of the objective acoustical properties of bowel sounds, particularly their significance during the perioperative period.^3^ Currently, clinical workers rely only on subjective measures such as auscultation of bowel sounds, abdominal circumference, and stool patterns.

Recently, a unique prototype system was developed jointly by industry and the tertiary education sector for the electronic monitoring and continuous assessment of bowel sounds based on noninvasive technologies that measure abdominal mechano‐acoustic activity in real time.^4^ In the present study, we prospectively evaluated bowel sound counts using this newly developed analysis system in patients who underwent gastric surgery.

## Methods

### *Patients*

This study was a prospective, single‐arm, observational study. We enrolled 14 patients with gastric tumor who underwent gastric surgery at Kochi Medical School during the period from January 2017 to December 2018. The eligibility criteria for patient inclusion in this study were as follows: patients undergoing gastric surgery, a performance status of 0–2 according to the Eastern Cooperative Oncology Group (ECOG) scale, aged 20 years or over, and expected to survive for more than 3 months from registration. Exclusion criteria were intestinal palsy, small bowel obstruction, renal failure, liver cirrhosis; massive ascites beyond the pelvic cavity or pleural effusion; women who were pregnant or hoped to become pregnant during the study period and men who wish their partner to become pregnant. Fourteen patients were included in the study analysis.

The study was approved by the Institutional Review Board at the Kochi Medical School Hospital (approval number: 29‐106), and was undertaken in accordance with the Helsinki Declaration and the Japanese Good Clinical Practice Guidelines. Informed consent was obtained from all individual participants included in the study.

### *Real‐time bowel sound analysis system*

**Figure 1:**
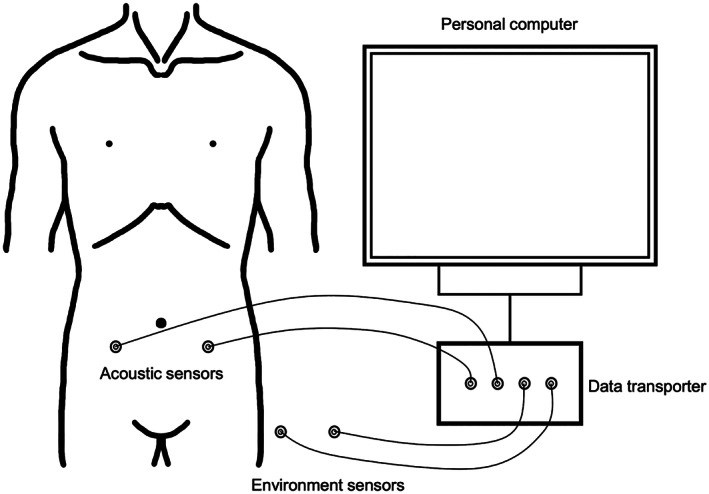
Schematic diagram of the real‐time, bowel sound analysis system. It consisted of four sensors with a multichannel data logger, an isolation transformer, a personal computer with the analysis software, and acoustic sensors.

The real‐time bowel sound analysis system consisted of recording equipment and acoustic sensors (Fig. 1). The recording equipment (Asahi Kasei Medical Co., Tokyo, Japan) consisted of four sensors with a multichannel data logger, an isolation transformer, and a personal computer with the relevant analysis software to detect and record bowel sounds in real time. The acoustic sensors comprised silicone‐covered, rectangular microphones with built‐in amplifiers. With these acoustic sensors attached to the abdomen, bowel sounds were counted if they matched a template power spectrum established in a previous study using normal bowel sound data.^5^, ^6^ The bowel sound counts per minute (cpm) were displayed in real time.

The patients were fasting for solid and liquid food 12 h before entering the operating theater, and general anesthesia was performed using sevoflurane or desflurane, with or without remifentanil hydrochloride, propofol, or the epidural anesthesia. The bowel sound count was recorded using this system during preoperative and postoperative periods in the operating theater and recovery room.

### *Statistical analysis*

We tested differences between mean values for the two groups of patients for significance by the Mann–Whitney *U* test for continuous variables and Pearson's Chi‐square test for categorical variables. Correlation between the bowel sound count and the operation time was evaluated by calculating Pearson's product‐moment correlation coefficient. Statistical analyses were performed using SPSS for Windows, version 22.0. Various factors to be tested by multivariate analysis were dichotomized according to the univariate analysis.

## Results

### *Patient characteristics*

Table 1 summarizes the clinical characteristics of patients who underwent gastric surgery using the real‐time bowel sound analysis system in the present study. The study cohort comprised 10 men and 4 women with a median age of 75 years (range, 44–91 years). The diagnosed gastric diseases were gastric cancer in 12 patients and gastrointestinal stromal tumor in 2 patients. Surgical treatment comprised total gastrectomy in eight patients, distal gastrectomy in four patients, and local resection of the stomach in two patients. The surgical approach was open gastrectomy in eight patients and laparoscopic gastrectomy in six patients. Median operation, preoperative, and postoperative times were 264.1 (range, 98.6–531.0), 27.8 (10.7–53.4), and 41.3 min (10.6–1314.6), respectively.

**Table 1:** Clinical characteristics of the study patients (*n* = 14)

Age (years), range	75 (44–91)
Gender	
Male	10
Female	4
Gastric diseases	
Gastric cancer	12
Gastrointestinal stromal tumor	2
Surgical method	
Total gastrectomy	8
Distal gastrectomy	4
Local resection of the stomach	2
Surgical approach	
Open	8
Laparoscopic	6
Median operation time (min), range	264.1 (98.6–531.0)
Median preoperative time (min), range	27.8 (10.7–53.4)
Median postoperative time (min), range	41.3 (10.6–1314.6)

### *Change in bowel sound count before and after surgery*

Table 2 summarizes the changes in bowel sound count before and after surgery, with median preoperative and postoperative counts across all patients recorded as 1.4 and 2.5 cpm, respectively. Although this difference overall did not reach significance (*P* = 0.258), the median postoperative bowel sound count for patients undergoing laparoscopic gastrectomy was significantly higher than the preoperative value (2.3 vs. 1.6 cpm, *P* = 0.005). There were no significant differences in the postoperative to preoperative bowel sound count ratio depending on the surgical method or surgical approach.

**Table 2:** Change in bowel sound count before and after surgery

	Preoperative	Postoperative	*P* value	Postoperative to preoperative BSC ratio	*P* value
All procedures (median, cpm)	1.4	2.5	0.258	1.79	
Median time (min)	27.0	41.3	0.070	1.53	
Surgical method					0.951
Total gastrectomy (median, cpm)	0.9	2.5	0.099	2.78	
Distal gastrectomy (median, cpm)	1.4	2.8	0.482	2.0	
Local resection of the stomach (median, cpm)	2.6	2.1	0.844	0.81	
Surgical approach					0.143
Open gastrectomy (median, cpm)	1.3	2.5	0.140	0.77	
Laparoscopic gastrectomy (median, cpm)	1.6	2.3	0.005	0.48	

*P* value significant at 0.05.

BSC, bowel sound count; cpm, count per minute.

### *Relationship between bowel sound count and operation time*

**Figure 2:**
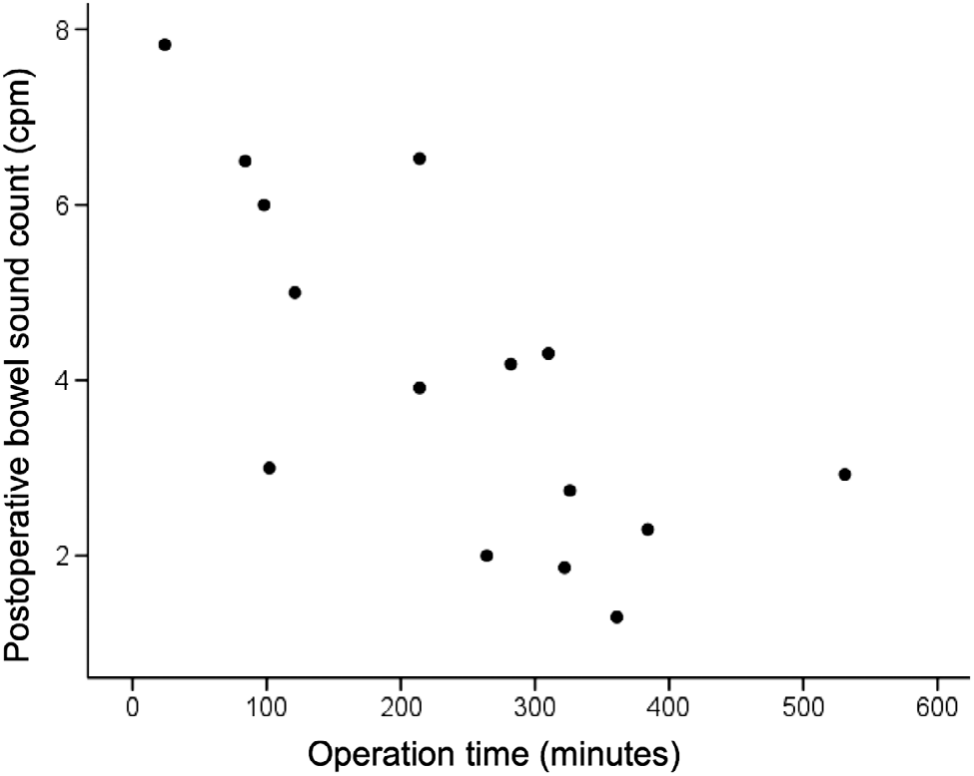
Scatter plot of the postoperative bowel sound count compared to operation time. A significant negative correlation was observed between the groups (*r* = −0.714, *P* = 0.003).

**Figure 3:**
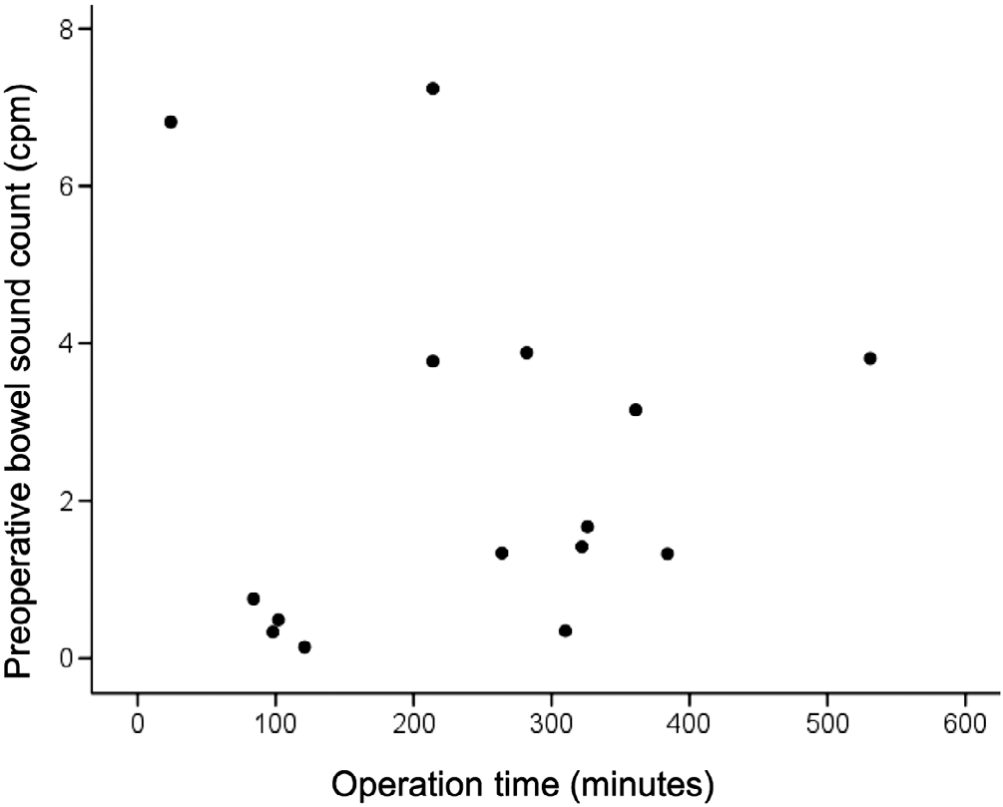
Scatter plot of the preoperative bowel sound count compared to operation time. No significant correlation was observed between the groups (*r* = 0.014, *P* = 0.960).

**Figure 4:**
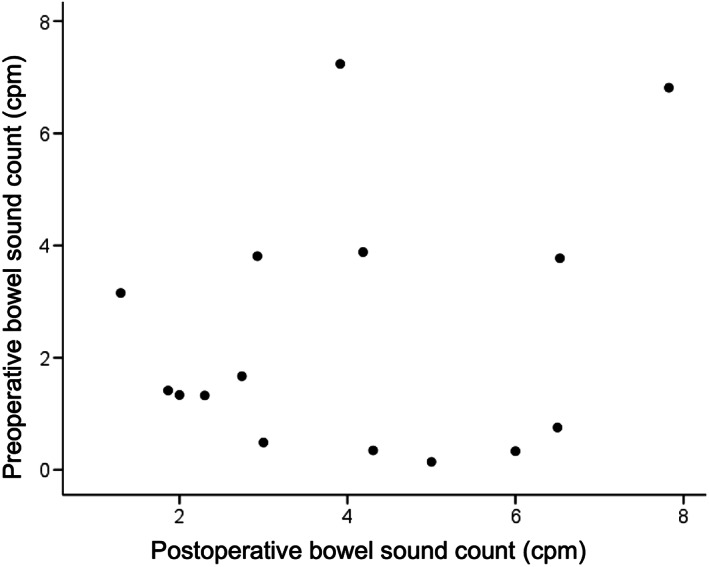
Scatter plot of the bowel sound count during the preoperative and postoperative periods. No significant correlation was observed between the groups (*r* = 0.227, *P* = 0.415).

Correlation between the bowel sound count and the operation time was evaluated. A significant negative correlation was identified between the postoperative bowel sound count and the operation time (*r* = −0.714, *P* = 0.003; Fig. 2). There was no significant relationship between the preoperative bowel sound count and the operation time (*r* = 0.014, *P* = 0.960; Fig. 3), and between the preoperative and postoperative bowel sound count (*r* = 0.227, *P* = 0.415; Fig. 4).

## Discussion

In this prospective study, we demonstrated quantitative results for perioperative bowel sounds obtained in real‐time that provide objective interpretations of how the acoustic activities of the intestine could be associated with motility. The main finding in our cohort of patients who underwent gastric surgery was an association between bowel sound count and operation time. While several studies have suggested the auscultatory assessment of bowel sounds is useful for detecting paralytic ileus with a strong positive predictive value,^7^, ^8^ there is no correlative evidence based on a quantitative evaluation of bowel sounds. Compared to traditional auscultation, the present study provides an important advantage that the result of bowel sound is recordable and easily comparable between different patients, or different tests on the same patient under postoperative monitoring. This is the first study to quantitatively assess the perioperative bowel sound count in patients undergoing surgery.

The assumption that bowel sound generation depends on the motility and mechanical properties of the intestine has remained the rationale for correlating a relationship between measurable differences in sounds heard through a stethoscope and the condition of the bowel.^9^ Relying on this premise, a clinical impression of the acoustic characteristics of bowel sounds by auscultation has been considered a surrogate for underlying gastrointestinal motility.^3^ Nevertheless, several studies question the usefulness of abdominal auscultation in patients with suspected bowel obstruction,^3^, ^10^ with Breum et al.^10^ showing low accuracy and interobserver agreement when recorded bowel sounds from 98 patients with possible bowel obstruction were assessed by 53 doctors. Furthermore, there is no evidence that listening to bowel sound is clinically useful in postoperative abdominal surgery patients. Our present results indicate that a new system for bowel sounds assessment that noninvasively visualizes and quantifies gastrointestinal acoustics in real time might provide an objective and useful analysis of intestinal motility.

To now, physicians auscultated patients with suspected intestinal obstruction and evaluated bowel sounds as either normal or pathological. Variation in the threshold used for abnormality is thus also an important factor in explaining the difference between normal and pathological bowel sounds. The advanced technology explored in this study allows for a more objective analysis of bowel sounds, using spectral analysis of the sounds together with an electronic bowel sound acoustic stethoscope.^2^, ^3^ Ching et al.^2^ also showed objectively that bowel sound characteristics were not significantly different among patients with acute, subacute, or no intestinal obstruction using the commonly compared parameters of bowel sound, including sound duration, sound‐to‐sound interval, and dominant and peak frequencies.

As a result of the present study, postoperative bowel sound was higher than preoperative bowel sound; however, the difference was not statistically significant. Although the intestinal peristalsis may recover relatively early after surgery, a verification study with a sufficiently large number of cases is necessary. Patients undergoing abdominal surgery were thought to experience reduced gastrointestinal peristalsis owing to extensive dissection, postoperative exhaust, and long duration of anesthesia.^5^ General anesthesia using both opioids and hypnotic agents such as volatile anesthetics might influence small bowel peristalsis, and although it is generally accepted that opioids induce intestinal paralysis, little is known about the effect of volatile anesthetics on intestinal motility.^11^, ^12^ Indeed, postoperative ileus, which is an interruption of bowel function after surgery, is a common sequela after bowel resections and other intra‐abdominal operations.^13^, ^14^ In addition, several studies demonstrated that chewing sugar‐free gum resulted in an earlier return to bowel function and lower analgesic requirements,^5^, ^14^, ^15^, ^16^ and that postoperative time to first bowel movement was significantly shortened by treatment with mosapride citrate.^17^ Real‐time bowel sound analysis system might, therefore, also be effective in quantitatively evaluating these pathophysiologies.

In the present study, there were no significant differences in postoperative to preoperative bowel sound count depending on surgical method and surgical approach. From these results, it remains unclear whether the bowel sound count is associated with the degree of surgical invasion. However, the postoperative bowel sound count in laparoscopic gastrectomy patients was significantly higher than the preoperative count. Hiki et al.^18^ demonstrated that inflammatory changes in the intestine, portal venous blood, liver, and systemic circulation were significantly upregulated by conventional open surgery compared with laparoscopic gastrectomy or even open gastrectomy without surgical manipulation of the intestines. Minimally invasive surgery including laparoscopic gastrectomy could, therefore, also contribute to fast recovery of intestinal motility.

The Enhanced Recovery After Surgery (ERAS) Society designed a multimodal perioperative care pathway to achieve earlier recovery after abdominal surgery and reduce the length of hospital stays.^19^, ^20^ The ERAS adaptions such as early postoperative feeding are considered effective in stimulating bowel movement, thereby possibly reducing postoperative ileus.^15^, ^20^ Although the ERAS concept is important for achieving faster recovery of gastrointestinal motility and improving clinical recovery, the measurements of postoperative bowel activity such as the time to flatus and defecation remain overly subjective.^20^ The bowel sound analysis system tested in this study might also prove useful to assess the multiple factors thought to exacerbate the pathogenesis of postoperative paralytic ileus, because the results are objective.

In the present bowel sound analysis system, portability of devise is limited due to its size including recording equipment including sensors with a multichannel data logger, an isolation transformer, and a personal computer. Currently, it can only be used in the hospital; however, the development of more compact analyzing systems might result in the use for outpatients or in long observation period.

There were several limitations in the present study. First, this study was conducted in a single institution with a relatively small number of subjects, and thus it could be affected by patient selection bias. As a matter of fact, significant variables on multivariate analysis were not found in this study. Second, this was a single‐arm, prospective observation study, and the fact that this was not a randomized control study could lead to selection bias. Third, the subjects in this study were limited to gastric surgery to exclude surgery, which directory invades the intestines. Therefore, the results of this study should be interpreted cautiously. Further studies with adequate statistical power and a larger number of patient subgroups are needed to determine the reliability and accuracy of using real‐time bowel sound analysis during the perioperative period to quantitatively evaluate and assess bowel movement.

In conclusion, a real‐time bowel sound analysis system seems promising as a diagnostic tool to quantitatively evaluate bowel movement. In this study, analysis of bowel sounds in patients undergoing gastrointestinal surgery suggested a need for shorter operation times from the aspect of peristalsis recovery and supporting the use of minimally invasive surgery. Further studies are still needed to confirm and update distinct and feasible standards regarding bowel sound analysis and results interpretation.
